# Bullous pemphigoid: epidemiological, clinical, and therapeutic analysis of 189 patients in a tertiary center in Brazil^☆^

**DOI:** 10.1016/j.abd.2025.501163

**Published:** 2025-07-18

**Authors:** Cecília Mirelle Almeida Honorato, Claudia Giuli Santi, Celina Wakisaka Maruta, Valeria Aoki, Denise Miyamoto

**Affiliations:** Department of Dermatology, Hospital das Clinicas, Faculdade de Medicina, Universidade de São Paulo, São Paulo, SP, Brazil

**Keywords:** Autoimmune diseases, Brazil, Bullous pemphigoid

## Abstract

**Background:**

Bullous pemphigoid (BP) is the most prevalent autoimmune bullous dermatosis with increasing incidence globally. There is a lack of literature on BP in the multiethnic Brazilian population.

**Objectives:**

To assess the epidemiological, clinical, and therapeutic characteristics of BP patients in a tertiary center in Brazil.

**Methods:**

Retrospective longitudinal review of clinical records of 189 BP patients from January 1986 to September 2023.

**Results:**

BP primarily affected elderly individuals, predominantly females, with an average onset of symptoms at 65.7-years. Non-bullous presentations had a longer time to diagnose compared to the bullous form. Mucosal involvement was observed in 24.9% of patients. Subepidermal blistering was the predominant histopathological feature. Most cases presented fluorescence of IgG and C3 at the basement membrane zone (BMZ) on direct immunofluorescence. Indirect immunofluorescence mainly revealed fluorescence of IgG along the BMZ, and with salt-split skin technique demonstrated predominantly IgG fluorescence on the epidermal side of the cleavage. Eosinophilia, elevated IgE levels, and D-dimer were common. Systemic corticosteroids remained the mainstay of treatment. BP was associated with significant complications, including thromboembolism, hospitalization, and infections, along with numerous comorbidities and a notable percentage (10.6%) of patients using potentially BP-inducing medications.

**Study limitations:**

The study's limitations include its retrospective design, reliance on potentially incomplete clinical records, and findings of a single tertiary center.

**Conclusions:**

This study provides crucial insights into the multifaceted nature of BP in the Brazilian population, emphasizing the need for comprehensive management strategies to address its diverse complications and associated conditions.

## Introduction

**Figure 1:**
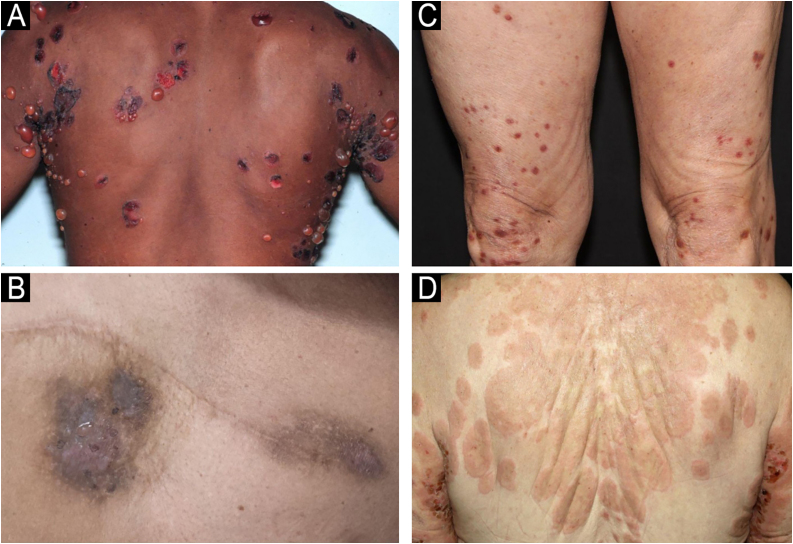
Clinical manifestations of classic BP with tense blisters (A) and non-bullous BP forms, including eczematous (B), prurigo-like (C), and urticariform (D).

Bullous pemphigoid (BP) is the most common autoimmune bullous dermatosis, mainly affecting elderly individuals over 70-years of age. There has been a significant increase in incidence over the last two decades due to population aging, earlier recognition of non-bullous forms of the disease, and increased availability of diagnostic methods. BP results from the production of autoantibodies against the hemidesmosomal antigens BP-180 and BP-230, which are components of the basement membrane zone (BMZ). The subsequent loss of adhesion between the epidermis and dermis leads to a subepidermal detachment resulting in tense blisters. This clinical presentation is classified as bullous or classic BP. However, other forms of the disease have been described, such as lichen planus pemphigoid and even non-bullous forms of BP, including prurigo-like, eczematous, dyshidrosiform, urticariform, and erythroderma.^1^, ^2^ Fig. 1 shows the clinical manifestations of classic BP and non-bullous forms, including eczematous, prurigo-like, and urticariform presentations.

There is a paucity of literature on BP in Brazil, a multiethnic country with an aging population. To our knowledge, this is the first study on the profile of BP patients in Brazil with a 37-year follow-up. The aim of the present study is to retrospectively evaluate the epidemiological, clinical, and therapeutic profiles of patients with BP followed at the Department of Dermatology, Hospital das Clinicas HCFMUSP, Faculdade de Medicina, Universidade de São Paulo, Brazil.

## Methods

This retrospective, longitudinal, and descriptive study included patients evaluated from January 1986 to September 2023 at the Department of Dermatology, Hospital das Clinicas HCFMUSP, Faculty of Medicine, Universidade de Sao Paulo, Brazil. After ethics committee approval (CAAE #63382922.2.0000.0068), patients with BP confirmed according to the following diagnostic criteria were included: compatible (1) clinical and (2) histopathological features, and the (3) presence of IgG and/or C3 at the BMZ demonstrated by Direct Immunofluorescence (DIF) and/or Indirect Immunofluorescence (IIF) with IgG deposits at the BMZ, and IIF with Salt-Split Skin (SSS) technique with IgG on the epidermal or epidermal and dermal side of the cleavage.

Medical records were reviewed to gather data on epidemiology, clinical presentation, comorbidities, histopathological and immunofluorescence findings, treatments administered, and complications. Patients with incomplete medical records that hindered the collection of important research data were excluded.

## Results

A total of 189 patients were included in the study, with a mean follow-up of 55-months. There was a female predominance (62.4%) and the majority of patients were White (85.2%). The mean age at onset of symptoms was 65.7-years, and the overall mean time to diagnosis was 9.3-months (6.5-months for bullous BP vs. 43.4-months for non-bullous BP). Two cases began during pregnancy.

BP with blisters was the most common initial presentation (88.3%), followed by the non-bullous forms (7.9%), among which 46.7% developed blisters after an average of 31.6-months. Mucosal involvement occurred in 24.9% of the patients, predominantly affecting the oral mucosa (95.7%). BP was one of the initial diagnostic hypotheses in 85.2% of cases, and the main differential diagnoses ‒ acquired epidermolysis bullosa (29.1%); linear IgA bullous dermatosis (14.8%); dermatitis herpetiformis (10.6%) ‒ were ruled out according to the immunofluorescence studies.

Cardiovascular and metabolic comorbidities were present in 77.8% of the patients, followed by neuropsychiatric conditions (25.4%), other autoimmune and inflammatory diseases (24.9%) and malignancies (23.3%). Potentially BP-inducing medications were observed in 10.6% cases: aldosterone antagonists (3.2%), Dipeptidyl Peptidase 4 (DDP4) inhibitors (3.2%), anticholinergics (2.6%), and dopaminergic medications (1.6%).

The main histopathological characteristics observed were subepidermal blistering (88.0%), eosinophilic spongiosis (6.0%), presence of eosinophils (52.2%), both eosinophils and neutrophils (35.3%), and exclusively neutrophils (4.9%). Major DIF findings included immune complex deposition at the BMZ with both IgG and C3 (73.8%), only C3 (20.8%) or IgG (5.5%). IIF with the SSS technique demonstrated IgG fluorescence on the epidermal side of the cleavage (81.9%) or both on the epidermal and dermal sides (4.0%). Additional assessment of the patients revealed elevated serum IgE levels (47/63; 74.6%), eosinophilia (91/87; 48.7%), and increased D-dimer levels (26/28; 92.8%).

Systemic treatment was the mainstay of therapy, with the use of corticosteroids (82.5%), doxycycline/tetracycline (44.9%), immunosuppressants (22.7%); dapsone (17.5%), rituximab (2.1%) and plasmapheresis (0.5%). Exclusive topical corticosteroid was used in 6.9% of patients and one patient experienced spontaneous remission. Data from the last consultation revealed that 40.7% of patients were in complete remission with therapy, and 30.7% were in complete remission off therapy. Partial remission with medication was observed in 22.2% of patients, and 5.8% experienced recurrence (>3-lesions lasting ≥7-days).

Venous thromboembolic events occurred in 7.9% of patients including deep vein thrombosis (72.2%), pulmonary embolism (22.2%) and portal vein thrombosis (5.5%). Secondary bacterial infection was experienced by 62.4% of patients. Hospitalization was required by 55.0% of patients either due to disease severity or infectious complications.

Data from 15 deaths was available, with infection as the leading cause (40.0%) followed by complications related to neoplasms (13.3%). The average disease duration at the time of the patient's death was 37.5-months. The main characteristics of the studied cases are described in Tables 1‒4 .

**Table 1:** Main epidemiological and clinical aspects of the studied cases of BP.

**Subjects (n)**		189
**Mean follow-up time (months), ± standard deviation**		55.0 (0.3 to 448)
**Sex**	Female	62.4% (118/189)
	Male	37.6% (71/189)
**Race/Ethnics**	White	85.2% (161/189)
	Multiracial	7.9% (15/189)
	Asian	3.7% (7/189)
	Black	2.1% (4/189)
	Not specified	1.0% (2/189)
**Mean age of onset (years), ± standard deviation**		65.7 (0.2 to 93)
**Mean time to diagnosis (months), ± standard deviation**	Overall	9.3 (0.2 to 221)
	Classic BP	6.5 (0.2 to 120)
	Non-bullous BP	43.4 (1 to 221)
**Clinical presentation at initial assessment**	Bullous	88.3% (167/189)
	Non-bullous	7.9% (15/189)
	Eczematous	40.0% (6/15)
	Prurigo-like	26.7% (4/15)
	Urticariform	13.3% (2/15)
	Erythroderma	13.3% (2/15)
	Dyshidrosiform	6.7% (1/15)
	Lichen planus pemphigoid	0.5% (1/189)
	No active lesions	2.1% (4/189)
	Not specified	1.0% (2/189)
**Mucosal involvement**	Total of patients with mucosal involvement	24.9% (47/189)
	Oral	95.7% (45/47)
	Nasal	10.6% (5/47)
	Larynx	8.5% (4/47)
	Esophageal	2.1% (1/47)
	Pharynx	2.1% (1/47)
	Conjunctival	2.1% (1/47)
**Pruritus**	Present	70.4% (133/189)
	Absent	0.0% (0/189)
	Not specified	29.6% (56/189)
**Main diagnostic hypotheses at initial assessment**	Bullous pemphigoid	85.2% (161/189)
	Acquired epidermolysis bullosa	29.1% (55/189)
	Linear IgA bullous dermatosis	14.8% (28/189)
	Dermatitis herpetiformis	10.6% (20/189)
**Potential BP-inducing medications**	Percentage of patients in use of one of them	10.6% (20/189)
	Aldosterone antagonists	3.2% (6/189)
	Dipeptidyl peptidase 4 inhibitors	3.2% (6/189)
	Anticholinergics	2.6% (5/189)
	Dopaminergic medications	1.6% (3/189)
**Complications**	Hospitalization rate	55.0% (104/189)
	Venous thromboembolism	7.9% (15/189)
	Secondary bacterial infection (at least 1 episode)	62.4% (118/189)
**Death**	Infections	66.7% (10/15)
	Complications associated with neoplasms	13.3% (2/15)
	Others (complications from an epigastric hernia, acute pulmonary edema, and myocardial infarction)	20.0% (3/15)

**Table 2:** Comorbidities presented by the patients.

**Cardiovascular and metabolic diseases**	77.8% (147/189)
Systemic arterial hypertension	63.5% (120/189)
Diabetes mellitus	38.6% (73/189)
Dyslipidemia	26.4% (50/189)
Osteoporosis	20.6% (39/189)
Chronic kidney disease	10.0% (19/189)
Congestive heart failure	9.5% (18/189)
Coronary artery disease	9.0% (17/189)
Arrhythmia	7.9% (15/189)
Gout	1.6% (3/189)
**Neurologic and psychiatric diseases**	25.4% (48/189)
Cerebrovascular disease	12.2% (23/189)
Dementia	9.5% (18/189)
Alzheimer's disease	4.8% (9/189)
Vascular dementia:	0.5% (1/189)
Mixed dementia	0.5% (1/189)
Unknown cause	3.7% (7/189)
Depression	6.9% (13/189)
Anxiety	3.2% (6/189)
Epilepsy	2.6% (5/189)
Parkinson's disease	2.6% (5/189)
Schizophrenia	1.6% (3/189)
Intellectual disorder	1.0% (2/189)
Bipolar affective disorder	0.5% (1/189)
Personality disorder	0.5% (1/189)
**Malignancies**	23.3% (44/189)
Skin	7.4% (14/189)
Basal cell carcinoma	5.3% (10/189)
Squamous cell carcinoma	3.2% (6/189)
Mycosis fungoides	0.5% (1/189)
Not specified	1.0% (2/189)
Prostate	3.2% (6/189)
Breast	2.6% (5/189)
Hematological	2.6% (5/189)^a^
Oral	1.0% (2/189)
Ovary	1.0% (2/189)
Stomach	1.0% (2/189)
Rectum	1.0% (2/189)
Others	5.3% (10/189)^b^
**Autoimmune and inflammatory diseases**	24.9% (47/189)
Hypothyroidism	11.1% (21/189)
Rheumatoid arthritis	5.8% (11/189)
Asthma	4.2% (8/189)
Psoriasis	1.6% (3/189)
Lupus erythematosus	1.0% (2/189)^c^
Allergic rhinitis	1.0% (2/189)
Psoriatic arthritis	0.5% (1/189)
Atopic dermatitis	0.5% (1/189)
Inflammatory bowel disease	0.5% (1/189)
Vitiligo	0.5% (1/189)
Lichen sclerosus	0.5% (1/189)
**Others**	
Immunosuppressive conditions	1.6% (3/189)
Renal transplant	0.5% (1/189)
Liver transplant	0.5% (1/189)
Common variable immunodeficiency	0.5% (1/189)
Ophthalmological	7.9% (15/189)
Cataract	4.8% (9/189)
Glaucoma	4.2% (8/189)
Infectious diseases	3.2% (6/189)
Hepatitis B	1.0% (2/189)
Hepatitis C	1.0% (2/189)
HIV	0.5% (1/189)
Tuberculosis	0.5% (1/189)

a Including myelodysplastic syndrome, multiple myeloma, myelofibrosis, lymphoma, and not specified (1-patient each).

b Including vulva, kidney, larynx, liver, lung, bladder, endometrium, intestine, brain, and not specified (1-patient each).

c Including one case of systemic lupus erythematosus and one case of discoid lupus.

**Table 3:** Main laboratory findings of the studied cases of BP.

**Histopathological findings**	
Subepidermal blistering	88.0% (162/184)
Eosinophilic spongiosis	6.0% (11/184)
Intraepidermal blistering	14.1% (26/184)
Eosinophils (without neutrophils)	52.2% (96/184)
Neutrophils (without eosinophils)	4.9% (9/184)
Eosinophils and neutrophils	35.3% (65/184)
**Direct immunofluorescence**	
Combined deposition IgG and C3 at the BMZ	73.8% (135/183)
C3 deposition at the BMZ (without IgG)	20.8% (38/183)
IgG deposition at the BMZ (without C3)	5.5% (10/183)
IgA deposition at the BMZ	9.3% (17/183)
IgM deposition at the BMZ	6.5% (12/183)
Negativity for IgG and C3	0.0% (0/183)
Inadequate sampling	1.6% (3/189)
Exam not performed	1.6% (3/189)
**Indirect immunofluorescence**	
IgG deposition at the BMZ	72.7% (120/165)
IgM deposition at the BMZ	2.7% (4/145)
IgA deposition at the BMZ	2.7% (4/146)
**Indirect immunofluorescence with salt-split skin technique**	
IgG on the epidermal side of the split	81.9% (122/149)
IgG on the epidermal and dermal side of the split	4.0% (6/149)
IgA on the epidermal side of the split	12.0% (18/150)
IgM on the epidermal side of the split	3.7% (2/54)
**IgE levels**	
Above the laboratory reference range	74.6% (47/63)
**Eosinophilia**	
Eosinophil count ≥ 500	48.7% (91/187)
**D-dimer levels**	
Above the laboratory reference range	92.8% (26/28)

**Table 4:** Therapies utilized by the patients during the follow-up period and their status at the final evaluation.

**Treatment** (percentage of patients who underwent each therapy during follow-up)	
Corticosteroid intralesional injection	2.6% (5/189)
Tetracycline	9.5% (18/189)
Doxycycline	35.4% (67/189)
Dapsone	17.5% (33/189)
Systemic corticosteroids	82.5% (156/189)
Methotrexate	7.4% (14/189)
Mycophenolate mofetil	11.1% (21/189)
Azathioprine	4.2% (8/189)
Rituximab	2.1% (4/189)
Plasmapheresis	0.5% (1/189)
**Outcome at the last consultation**	
Complete remission with medication	40.7% (77/189)
Complete remission without medication	30.7% (58/189)
Partial remission with medication	22.2% (42/189)
Partial remission without medication	0.5% (1/189)
Recurrence (>3-lesions lasting ≥7-days)	5.8% (11/189)

## Discussion

BP is the most common autoimmune blistering dermatosis, primarily affecting individuals of white race over 70-years of age, with no gender predilection.^2^ In this case series, the disease primarily affected elderly individuals with a mean age of symptom onset of 65.7-years, mostly white (85.2%), and with a slight female predominance (62.4%). As a referral center, this case series included five rare cases of children with BP, which may have slightly lowered the average age. Notably, the ethnic composition of the sample contrasts with the 2022 Census data,^3^ where only 43.5% of the population self-identified as white. As this is a single-center study, this limitation may have contributed to the observed differences, reflecting regional characteristics and healthcare access disparities.

The overall average time for diagnosing BP was 9.3-months, longer in non-bullous than in bullous forms, which may occur because the absence of blisters can pose a challenge for clinicians in recognizing this condition.^1^ It is important to mention that patients referred to the tertiary center often have a history of multiple consultations in different healthcare facilities, leading to a delayed diagnosis. Notably, 46.7% of patients with non-bullous BP developed blisters after a mean of 31.6-months, a higher proportion than previously reported,^1^ likely due to the extended follow-up (mean of 55.0-months).

In the present study, mucosal involvement was observed in 24.9% of patients (within the reported range of 10%‒30%),^1^, ^2^, ^4^ primarily in the oral mucosa, and was associated with classic BP and increased severity of cutaneous disease. Pruritus was a common symptom, highlighting the importance of considering BP as a potential differential diagnosis for chronic pruritus in elderly patients.^1^, ^2^ BP was one of the initial diagnostic hypotheses in 85.2% of cases, with the main differential diagnoses being other subepidermal blistering dermatoses.

**Figure 2:**
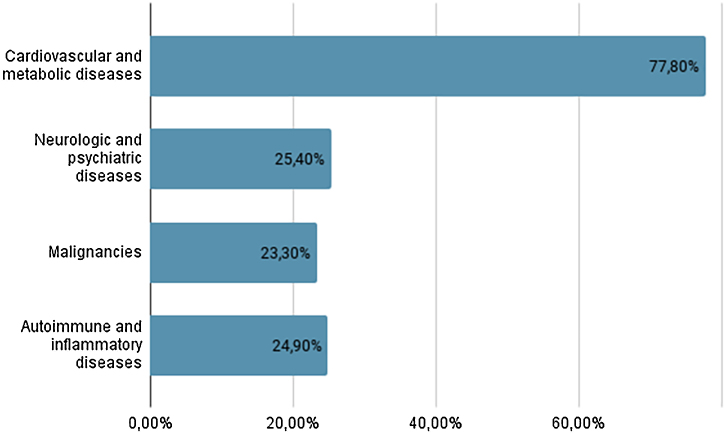
Graph showing the prevalence of the main disease groups presented by the patients.

Fig. 2 presents the prevalence of the main disease groups among patients. Neurological and psychiatric conditions were present in 25.4% of patients, and studies indicate that 26.4%‒55.8% of BP patients have at least one neurological condition.^5^ Cardiovascular and metabolic conditions were prevalent in 77.8% of patients, consistent with a higher prevalence of metabolic syndrome associated with BP,^5^ contributing to increased morbidity. The association between BP and malignancies remains controversial, but the high rate of this association (found in 23.3% of these cases) could be due to the prevalence of BP in older age groups.^5^ Autoimmune and inflammatory comorbidities were present in 24.9% of patients, reflecting the autoimmune nature of BP.^5^

A recent meta-analysis suggested a connection between certain medications, such as aldosterone antagonists, DPP4 inhibitors, anticholinergics, and dopaminergic drugs, and the development of BP.^6^ In the present study, 10.6% of patients were using at least one of these potentially BP-inducing medications at their initial assessment. Further analysis is needed to guide informed prescribing practices for BP management, as some of these medications are essential for treating comorbidities.

**Figure 3:**
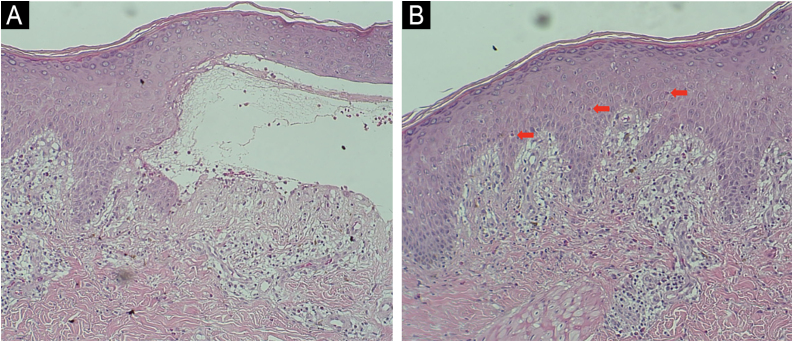
Skin biopsy: Histopathological findings of (A) subepidermal blistering with eosinophils in the classic BP form (Hematoxylin & eosin, ×4), and (B) presence of eosinophilic spongiosis (intraepidermal eosinophils within areas of spongiosis) in a case of non-bullous form (Hematoxylin & eosin, ×4).

Histopathological findings primarily showed subepidermal blistering, often accompanied by eosinophils and/or neutrophils, which is consistent with the typical findings of bullous lesions, while non-bullous lesions usually demonstrate the presence of eosinophilic spongiosis (Fig. 3). Intraepidermal blistering may be attributed to the phenomenon of re-epithelialization and also to significant spongiosis, leading to intraepidermal vesication.^7^

**Figure 4:**
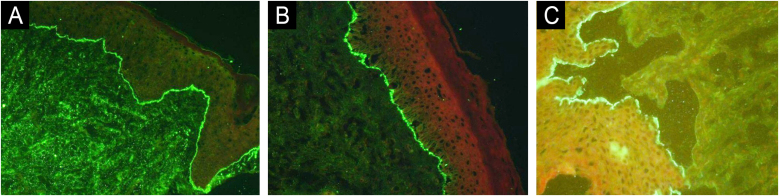
Immunofluorescence findings with DIF demonstrating IgG (A) and C3 (B) deposition at the BMZ, and IIF with salt-split technique demonstrating IgG deposition at epidermal side of the detachment (C).

DIF revealed combined IgG and C3 fluorescence at the BMZ in 73.8% of cases, with no negativity for both markers, indicating the high sensitivity of this method. IIF detected IgG deposits along the BMZ in 72.7% of cases, while SSS showed higher positivity, with 81.9% of cases presenting IgG fluorescence on the epidermal side ‒ an expected result given the higher sensitivity of the SSS technique.^8^ All three techniques demonstrated low positivity values for IgA and IgM. The positivity of these immunoglobulins is reported in the literature, with suggested associations between IgA positivity and mucosal involvement.^9^, ^10^, ^11^ Fig. 4 shows the main findings of DIF and SSS techniques.

A significant portion of the evaluated cases presented eosinophilia and elevated IgE and D-dimer levels. These markers correlate with BP severity and activity, serving as important prognostic indicators.^2^ However, the retrospective nature of the study limited further analysis, as these parameters were not available for all patients.

Immunosuppression is the primary treatment for BP, with only 6.9% of patients using topical corticosteroids alone, despite literature indicating their efficacy for mild cases.^2^ Systemic corticosteroids were administered to 82.5% of patients, likely due to the higher severity of cases at the tertiary center, and only one patient experienced spontaneous improvement without any treatment. However, despite the challenges in treating BP patients, the present study showed promising treatment outcomes, as most patients were in complete remission at their last evaluation, regardless of still required specific medication.

The hospitalization rate in these cases was 55.0%, aligning with previous reports of 53.0%.^12^ Venous thromboembolism occurred in 7.9% of patients, mostly after disease onset, likely due to BP-induced immune dysregulation, which promotes a pro-inflammatory state and increases thromboembolic risk.^2^ Additionally, 62.4% of patients experienced at least one episode of secondary bacterial infection, a common complication and major cause of hospitalization in BP patients.^13^ Infections, particularly pneumonia, were the leading cause of mortality, though mortality data were available for only 15 patients, representing a limitation.

## Conclusion

In conclusion, the complexities of BP are apparent, with diverse clinical presentations, associated diseases, challenging treatment regimens, and multiple complications. Despite its limitations, including a retrospective design and findings from a single tertiary center, this study provides crucial insights into the multifaceted nature of BP in the Brazilian context, emphasizing the need for a comprehensive approach to diagnosis, management, patient care, and prevention of complications.

## ORCID ID

Cecília Mirelle Almeida Honorato: 0009-0004-6837-1606

Claudia Giuli Santi: 0000-0003-3650-4254

Celina Wakisaka Maruta: 0000-0002-0541-5526

Denise Miyamoto: 0000-0002-4133-4475

## Financial support

None declared.

## Author's contribution

Cecília Mirelle Almeida Honorato: Critical literature review; data collection, analysis and interpretation; preparation and writing of the manuscript; statistical analysis; study conception and planning.

Claudia Giuli Santi: Approval of the final version of the manuscript; effective participation in research orientation; intellectual participation in propaedeutic and/or therapeutic management of studied cases; manuscript critical review; study conception and planning.

Celina Wakisaka Maruta: Approval of the final version of the manuscript; effective participation in research orientation; intellectual participation in propaedeutic and/or therapeutic management of studied cases; manuscript critical review.

Valeria Aoki: Approval of the final version of the manuscript; effective participation in research orientation; intellectual participation in propaedeutic and/or therapeutic management of studied cases; manuscript critical review.

Denise Miyamoto: Critical literature review; preparation and writing of the manuscript; study conception and planning; approval of the final version of the manuscript; effective participation in research orientation; intellectual participation in propaedeutic and/or therapeutic management of studied cases; manuscript critical review.

## Conflicts of interest

None declared.
